# Design and Analysis of Elimination Surveys for Neglected Tropical Diseases

**DOI:** 10.1093/infdis/jiz554

**Published:** 2020-01-13

**Authors:** Claudio Fronterre, Benjamin Amoah, Emanuele Giorgi, Michelle C Stanton, Peter J Diggle

**Affiliations:** Centre for Health Informatics, Computing and Statistics, Lancaster University, Lancaster, United Kingdom

**Keywords:** disease mapping, elimination surveys, geostatistics, neglected tropical diseases, predictions

## Abstract

As neglected tropical diseases approach elimination status, there is a need to develop efficient sampling strategies for confirmation (or not) that elimination criteria have been met. This is an inherently difficult task because the relative precision of a prevalence estimate deteriorates as prevalence decreases, and classic survey sampling strategies based on random sampling therefore require increasingly large sample sizes. More efficient strategies for survey design and analysis can be obtained by exploiting any spatial correlation in prevalence within a model-based geostatistics framework. This framework can be used for constructing predictive probability maps that can inform in-country decision makers of the likelihood that their elimination target has been met, and where to invest in additional sampling. We evaluated our methodology using a case study of lymphatic filariasis in Ghana, demonstrating that a geostatistical approach outperforms approaches currently used to determine an evaluation unit’s elimination status.

As neglected tropical diseases approach elimination status, there is a need to devise efficient sampling strategies to determine whether elimination criteria have been met. Before we can do this, we need to define what constitutes elimination. To do so requires decision makers to reach a consensus on (at least) the following considerations: (1) Over what geographic region, *A*, is elimination status to be determined? (2) Within *A*, at what spatial scale should elimination be assessed? (3) What diagnostic will be used to measure prevalence? (4) What critical prevalence threshold, *c*, represents elimination? and (5) What level of uncertainty associated with a declaration of elimination is acceptable?

In requirement 2 above, the spatial unit at which elimination is assessed is called an *evaluation unit* (EU). We emphasize that requirement 5 is inescapable. Absolute certainty can only be achieved by assessing disease status of the complete population using a perfect diagnostic, which is infeasible. We also argue that the conventional approach of *estimating* prevalence with an associated standard error or confidence interval does not directly address the decision maker’s problem. Instead, our methodology delivers, for each EU or any specified combination of EUs, the probability that the decision maker’s criterion (requirement 4 above) is met, given all available data.

In the following sections, we summarize the model-based geostatistics (MBG) approach to prevalence mapping, describe how the choice of sampling locations affects precision and explain why random sampling may not be the optimal strategy. We then discuss how to use pre-elimination (or “baseline”) data in conjunction with a simulation model for disease progression toward elimination status to design an elimination survey. We demonstrate our recommended design and analysis strategy with a case study on lymphatic filariasis (LF) mapping in Ghana.

## METHODS

### Geostatistical Modeling and Predictive Inference

A generic goal for disease mapping is to map the variation in prevalence over a finite set of *prediction locations*$x_{j}^{*}:j=1,...,N$ in a designated region *A*. Typically, the prediction locations will either be the known locations of all at-risk communities in *A* or a regular grid at a sufficiently fine spacing that, for practical purposes, can be taken to represent the whole of *A*. Critically for our purpose, any such map should be accompanied by a quantitative assessment of its associated uncertainty.

The MBG approach to problems of this kind rests on the following very general formulation of a prediction problem. The objective is to predict some property of the true, but unknown, state of nature, *S*, using data, *Y*, that convey partial information about *S*. The solution requires the user to formulate 2 stochastic models: a *process model* for *S*; and a *data model* for *Y* conditional on *S*. Bayes’ theorem then converts this into the distribution for *S* conditional on *Y*, called the *predictive distribution* of *S*, from which the predictive distribution of any property of *S* follows automatically.

Diggle and Giorgi [1] give a full account of MBG with applications in global public health research. Giorgi and Diggle [2] describe an open-source software implementation, the R package PrevMap, which can be downloaded from the CRAN repository on the R project website (www.r-project.org).

#### Geostatistical Model

Prevalence data typically consist of a set of geolocations *x*_*i*_:*i* = 1,…,*n* in the region of interest *A*; *m*_*i*_ people are tested at location *x*_*i*_, yielding *Y*_*i*_ positive tests. A vector of covariates, *d*(*x*), may also be available at each location. A geostatistical model for prevalence data assumes that, conditional on the state of nature *S*, the *Y*_*i*_ are independent binomial random variables with denominators *m*_*i*_ and probabilities *P*(*x*_*i*_) that a person at location *x*_*i*_ will test positive, where

$$\begin{array}{l} \log\left[P\left(x\right)/\left\{1-P\left(x\right)\right\}\right]=\alpha +d{\left(x\right)}^{\prime }\beta +S\left(x\right)+Z\left(x\right). \end{array}$$ (1)

In (1), *S*(*x*) and *Z*(*x*) are sets of random effects that account, respectively, for spatially structured and spatially unstructured variation in prevalence that is not explained by the covariates.

The standard formulation for the additional terms *S*(*x*) and *Z*(*x*), and the one we use here, is that both are normally distributed. The distinction between them is that for any 2 locations *x*_1_ and *x*_2_, the random variables *Z*(*x*_1_) and *Z*(*x*_2_) are independent, whereas *S*(*x*_1_) and *S*(*x*_2_) are generally correlated, with correlation ρ(*u*) that depends on the distance *u* between *x*_1_ and *x*_2_. When we use this model for predicting the prevalence surface *P*(*x*), the practical effect of the additional terms *S*(*x*) and *Z*(*x*) is to adjust the predictions of the prevalence surface *P*(*x*) that would have been delivered by a logistic model without random effects, to an extent determined by the strength and range of the spatial correlation exhibited by the data. The *Z*(*x*) term does not directly affect point prediction, in the sense that the best point prediction of *Z*(*x*) at an unsampled location *x* is 0, whereas the best point prediction of *S*(*x*) is in general non-0 because it takes account of data from nearby sampled locations. However, and importantly, both terms contribute to the uncertainty attached to the predictions. Moreover, inclusion of the *Z*(*x*) term prevents the fitted model from forcing spatial correlation when the data offer no support for this. In the Supplementary Materials we provide more technical details on this geostatistical model and the estimation of the model parameters.

#### Defining Predictive Targets for EUs

The geographic region *A* will usually be partitioned into a set of EUs, for each of which we wish to determine their elimination status. If the locations of communities in an EU are unknown, a natural target for prediction is the population-weighted average prevalence,

$$\begin{array}{l} T=\frac{\int P\left(x\right)\lambda \left(x\right)dx}{\int \lambda \left(x\right)dx}, \end{array}$$ (2)

where *P*(*x*) and λ(*x*) are the prevalence and population density, respectively, at *x* and the integral is over the whole of the EU in question. In practice, we replace the integral in (2) by a sum over a fine grid, using estimates of λ(*x*), for example WorldPop estimates (https://www.worldpop.org). If, as is the case for our Ghana example, the locations *x*_*j*_ and approximate numbers of inhabitants *n*_*j*_ for all communities in each EU are known, *T* reduces to a finite sum over all of the communities,

$$\begin{array}{l} T=\frac{\sum n_{j}P\left(x_{j}\right)}{\sum n_{j}}. \end{array}$$ (3)

The predictive target can also be modified to take account of known sensitivity, *Se*, and specificity, *Sp*, of the diagnostic test [3]. If *T* = *P*(*x*) denotes prevalence as measured by the diagnostic, and T* = P*(*x*) denotes true prevalence, then

$$\begin{array}{l} T=\left(1-Sp\right)+\left(Se+Sp-1\right)\times T^{*}. \end{array}$$

Under the reasonable assumption that *Se* + *Sp* > 1 (ie, that the test is superior to the toss of a coin), *T* is an increasing function of T* and the inverse relationship is

$$\begin{array}{l} T^{*}=\left\{T-\left(1-Sp\right)\right\}/\left(Se+Sp-1\right). \end{array}$$ (4)

If *Se* and *Sp* are not known exactly, it may be possible to assign to them a joint probability distribution, in which case drawing a sample from the predictive distribution of T* involves the following 3 steps: first, draw a sample from the predictive distribution of *T*; next, draw a sample from the joint distribution of (*Se,Sp*); finally, apply (4) to give the sampled value of T*.

### Geostatistical Design

A *geostatistical design* is a set of locations, $X=\left\{x_{i}\in A:i=1,\ldots ,n\right\}$ at which data will be collected. Typically, the value of *n* is limited by cost constraints, in which case the design question reduces to where to place the data locations.

Intuition suggests that a spatially regular arrangement of data locations should be most efficient. This turns out to be correct under 2 conditions: the underlying geostatistical model is known, and the predictive goal is spatially neutral; that is, predictive precision is equally important at all locations in *A*. Matérn [4] showed that under these circumstances, placing data locations at random is almost always inefficient, and in particular that lower mean square prediction errors can be achieved by a spatially regulated design of the kind illustrated in Supplementary Figure 2, which imposes a minimum permissible distance, δ, between any 2 sampled locations. When a design of this kind is applied to a finite set of potential sampling locations (for example, known villages within a designated region), not every potential location is equally likely to be included in the design; hence, simple summary statistics such as the sample mean, which ignore spatial correlation, are not unbiased estimates of their whole-population counterparts. Furthermore, the configuration of the potential locations may limit the ability to achieve the theoretical benefits of spatially regulated sampling.

When the predictive target is not spatially neutral, an adaptive design [5] can deliver more precise predictions. A 2-stage adaptive design consists of choosing a spatially neutral initial design with *m* < *n* data locations, constructing a predictive map from the resulting data and then adding the remaining *n ‒ m* data locations to optimize the design with respect to the predictive target of interest. For example, in an elimination survey, a reasonable goal is to establish whether, for each location in *A*, prevalence does or does not exceed *c*. The predictive target, *T,* is therefore the binary indicator of whether prevalence is less than *c*, and the second phase of the adaptive design can ignore areas where, based on the data from the initial design, the predictive probability *P*(*T* = 1|data) is sufficiently close to 0 or 1.

### Modeling Progression Toward Elimination

Any sample size calculation requires the analyst to assume a statistical protocol that will be applied to the data when they are collected. If the prospective data consist of independent random samples from one or more distribution, the required calculations are straightforward. In the current context, the required calculations are mathematically intractable and we therefore use simulation.

The simplest way to construct a model for evaluating the properties of a proposed elimination survey design is to use a model of the form (1) fitted to historic prevalence data, but with the parameter α scaled so as to lower the average prevalence to a value at, or close to, the agreed elimination level. In particular, to simulate data whose area-wide average prevalence is close to a particular value, ξ, we proceed as follows. First, let *P*(*x*) denote the predicted preintervention prevalence surface, and calculate the corresponding log-odds surface, L(x)=log[P(x)/{1−P(x)}]. Second, calculate *L*_0_(*x*) = *L*(*x*) − α _0_ and $P_{0}\left(x\right)=\exp\left\{L_{0}\left(x\right)\right\}/\left[1+\exp\left\{L_{0}\left(x\right)\right\}\right]$, where α _0_ is chosen so that the area-wide population-weighted average of *P*_0_(*x*) is ξ. Third, take a sample of villages and, within each sampled village, assign disease status to sampled individuals according to the value of the local prevalence, *P*_0_(*x*).

Sampling design evaluations could also use any stochastic model thought to be more appropriate for the disease in question. In principle, a well-informed disease-specific transmission model for progression from baseline to near-elimination status could provide more reliable simulations than a simple thinning of a geostatistical model; for examples of such models, see [6] or [7]. However, for our purpose, a major limitation of currently available models is that, although they are informed by a baseline prevalence map, their subsequent evolution to near-elimination status is not spatially explicit; that is, prevalences at different locations are assumed to evolve independently.

Whatever approach is used, once we have agreed on a model for the spatial variation in prevalence at or near-elimination status, then we can use simulations of the model to choose a sampling design that performs well against an agreed predictive target while incorporating specified practical constraints, and to show what sample size would be needed to achieve the required predictive precision. Practical constraints typically include the affordable sample size, perhaps tempered by considerations of the time taken to reach the complete set of sampled locations.

### A General Strategy

We propose that the design of an elimination survey should proceed as follows. First, agree on the area to be surveyed, and on a precise definition of the elimination target, *T*. Then use the best available pre-elimination data set to fit a geostatistical model of the pre-elimination spatial prevalence process. Next, use the fitted geostatistical model in conjunction with a model for progression toward elimination to simulate the spatial prevalence process at elimination. Finally, evaluate relevant performance characteristics of any candidate design by analyzing simulated data sets using MBG. In any specific context, turning this general approach into a specific design and analysis protocol will involve a number of disease-specific and local context-specific considerations. In the next section, we consider the assessment of LF elimination status in Ghana, comparing an emulation of the current transmission assessment survey (TAS) protocol with our proposed geostatistical approach.

### Simulation Study

We have carried out a simulation study to compare the efficiency of 2 survey designs and decision-making approaches for assessing LF elimination status in Ghana. In this setting, the survey design concerns the number and locations of statistical units (either communities/villages or schools) to sample and how many individuals to test in each unit.

Our study region *A* was the whole of Ghana, which is partitioned into 216 administrative districts. For the analysis reported here, each district acted as an EU. In our simulated data sets, only 164 of the 216 EUs passed a pre-TAS [8] (see also Supplementary Materials and Supplementary Figure 5) that determines their eligibility for the actual TAS, and we restrict our analyses to these 164 EUs.The criterion for elimination is that the EU-wide average prevalence of LF, as measured by an immunochromatographic test (ICT) to detect parasite antigen, is <2%.

We extracted data from the World Health Organization Expanded Special Project for Elimination of Neglected Tropical Diseases (ESPEN) portal [9] on 403 preintervention LF prevalence surveys conducted between 1998 and 2004 using the ICT diagnostic (Supplementary Figure 3). We also obtained the locations of 14 226 communities across Ghana using OpenStreetMap [10] and the GeoNames database [11] (Supplementary Figure 7). Because it was not possible to retrieve the numbers of people living in each community, we set these equal to the total population at EU level divided by the number of communities within that EU.

We first fitted the geostatistical model to the pre-elimination data, as described earlier and in the Supplementary Materials. To simulate a prevalence surface with country-wide average prevalence close to the elimination threshold of 2% ICT prevalence, we then adjusted the intercept parameter α in equation (1), as described earlier, holding the other model parameters fixed at their estimated preintervention values. We then simulated 100 prevalence data sets from this prevalence surface under each of the following design and analysis strategies.

Our first strategy was constructed to emulate the TAS protocol, by following World Health Organization guidelines [8]; see Supplementary Materials for a detailed description. This essentially specifies random sampling of communities within each EU in conjunction with a nonstatistical decision rule that compares the total number of sampled individuals in that EU who test positive for LF with a tabulated cutoff value [8]. Our other strategies used either a random or a spatially regulated sampling scheme within each EU in conjunction with a set of decision rules that declare elimination status for a EU if there is a sufficiently high predictive probability that its target *T*, as defined by equation (3), does not exceed the required 2%.

We summarize the performance of each strategy by its negative predictive value (NPV) and positive predictive value (PPV), averaged over the 164 TAS-eligible EUs. The PPV (NPV) expresses the probability that EUs where elimination has (has not) been declared have (have not) truly achieved elimination. An approach that can assess the elimination status of an EU with 100% accuracy would have both NPV and PPV equal to 1. In practice, there is always a trade-off between these values and policy makers’ need to carefully consider the programmatic implications of false-positives and false-negatives in an elimination context. For the TAS emulator, each of NPV and PPV is a single number. For any of the MBG strategies, we first calculate for each EU the predictive probability that *T* does not exceed 2%, and we declare elimination to be indicated if this probability is greater than a specified value, *q*. NPV and PPV are now each indexed by *q*, and we use a receiving operating characteristic curve to summarize overall performance.

We assessed a total of 12 MBG strategies by considering all combinations of the following: random or spatially regulated sampling within each EU; sampling the same number of communities as the TAS, one-fifth as many, or one-tenth as many; sampling the same total number of children or the same number of children per community as the TAS. For the spatially regulated designs we used the following rule to specify the minimum permissible distance, δ, between any 2 sampled communities within an EU. Let *d*_*j*_ be the distance from the *j*th community to its nearest neighbour and *n* the required number of communities to be sampled. Sort these distances in decreasing order and set the minimum permissible distance to δ = *d*_*n*_.

## RESULTS

**Figure 1.**
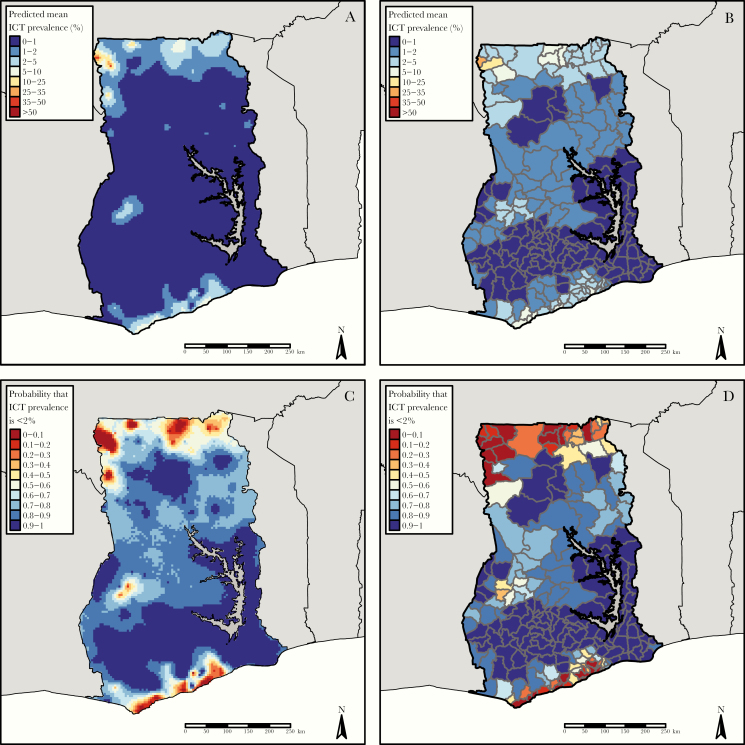
*A,* Predicted mean immunochromatographic test (ICT) prevalence at 5-km pixel level. *B*, Predicted mean population weighted ICT prevalence at the evaluation unit (EU) level. *C, D,* Probability maps for nonexceedance of 2% prevalence at pixel level (*C*) and nonexceedance of 2% population-weighted ICT prevalence at EU level (*D*).

Supplementary Table 1 shows parameter estimates and 95% confidence intervals for the geostatistical model fitted to baseline data, and Supplementary Figure 4 shows the predicted baseline prevalence surface. Figure 1A and1B summarize the predicted prevalence surface at elimination, *P*_0_(*x*), at 5-km pixel resolution and aggregated to the EU level.

For the spatially regulated sampling designs, the means (and ranges) of the minimum distance δ across the EUs were 1.67 (0.63–5.10), 3.84 (1.18–12.80), and 4.73 (1.35–17.22) km for designs using the same, one-fifth or one-tenth the TAS-specified number of locations per EU, respectively.

**Figure 2.**
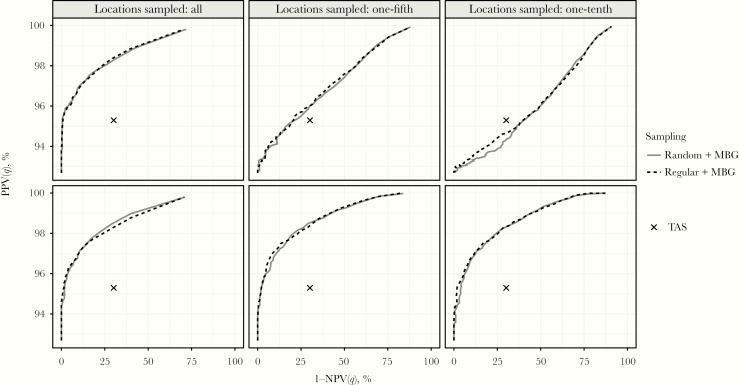
Receiving operating characteristic curves for the model-based geostatistics (MBG) approach applied to the spatially regulated (*dashed line*) and random (*solid line*) sampling schemes for 164 evaluation units in Ghana, with varying fraction (one-fifth or one-tenth) of locations sampled, compared with the transmission assessment survey (TAS) emulator. The number of children sampled per village is either the same as in the TAS (*upper row*) or increased relative to the fraction of villages sampled (*lower row*). The *x*’s indicate the performance of the TAS emulator; note that we do not change the fraction of villages or children sampled for the TAS. q is the probability threshold associated with the declaration of elimination for an evaluation unit (EU). That is, if the predictive probability that the population weighted prevalence does not exceed 2% is greater than q then elimination is declared. Abbreviations: NPV, negative predictive value; PPV, positive predictive value.

The mean PPV and NPV for the TAS emulator over the 164 EUs are 0.953 and 0.699. Figure 2 compares this with the performances of the MBG strategies using receiving operating characteristic curves. The MBG approach delivers better predictive performance than the TAS protocol, except in the extreme case of sampling one-tenth the number of communities and the same number of individuals per community as for the TAS protocol.

## Discussion

The simulation study has shown that accounting for spatial correlation and the incorporation of uncertainty in the assessment of elimination through an MBG framework can lead to significant gains in efficiency by comparison with the TAS protocol. Theoretical results suggest that a spatially regulated sampling scheme should be more efficient than random sampling when the underlying data generating process is spatially correlated. However, these results assume that sampling can take place at any location within the geographic region of interest. In practice, when the potential sampling locations are limited to a finite set the gains may be negligible if, as is the case in our Ghana LF example, the configuration of potential sampling locations constrains the minimum achievable distance between 2 sampled locations to a value substantially smaller than the theoretical optimum.

The efficiency of any sampling strategy is determined by the combination of the number of communities sampled and the number of individuals sampled per community. When we fix the number of individuals sampled per community, the MBG strategies outperform the TAS protocol when sampling the same or one-fifth the number of communities as the TAS, but not when sampling one-tenth the number of villages. In practice, the cost of sampling an extra community is likely to be substantially higher than the cost of sampling the equivalent number of extra children per community. For this reason, we also considered MBG strategies in which we fix the total number of individuals sampled to be the same as for the TAS. Under this scenario, the MBG strategies outperform the TAS protocol even when sampling one-tenth the number of villages, because the reduction in the binomial sampling variability within communities compensates for the smaller number of villages sampled.

In any particular application, the optimum balance between the number of communities sampled and the number of individuals sampled per community will depend on local cost considerations. Also, the extent to which an MBG strategy can improve on the TAS protocol will depend on the strength and range of the spatial correlation in the underlying prevalence surface. For the Ghana LF data, the estimated range of the spatial correlation is approximately 95 km, which is very large relative to the typical distance between neighbouring communities. This is exactly the situation in which exploiting the spatial correlation can deliver very substantial gains in efficiency.

In the MBG approach, the default model for the underlying true prevalence surface is an *empirical model*;that is, its parametric form is chosen to fit the observed pattern of spatial variability in the data. It is not informed by any scientific knowledge of the underlying disease transmission mechanism nor, conversely, does it make any assumptions about the underlying mechanism.

The simplest form of the geostatistical model for prevalence data, and the one used here, is that the log-odds of the prevalence surface *P*(*x*) is a realization of a Gaussian stochastic process. Equation (1) sets out a more general model that includes covariate effects in the linear predictor. Incorporating covariate information on variables that are associated with disease risk (eg, land cover, elevation, etc) can improve the precision of predictive inferences, provided that this information is available at every prediction location of interest. In practice, this usually requires each candidate covariate to be available as a raster image to cover the study region.

Incorporating well-founded disease-specific knowledge into a model for the spatial variation in prevalence can potentially “buy information with assumptions” [12]. This is especially relevant when seeking to establish compliance (or not) with very low prevalence thresholds for elimination, because this is exactly the situation in which the data themselves convey relatively imprecise information.

## Supplementary Data

Supplementary materials are available at *The Journal of Infectious Diseases* online. Consisting of data provided by the authors to benefit the reader, the posted materials are not copyedited and are the sole responsibility of the authors, so questions or comments should be addressed to the corresponding author.

jiz554_suppl_Supplemental_Material

## Notes

***Financial support.*** The NTD Modelling Consortium is supported by the Bill & Melinda Gates Foundation (grant OPP1184344).

***Supplement sponsorship.*** The authors acknowledge funding of the NTD Modelling Consortium by the Bill and Melinda Gates Foundation (OPP1184344).

***Potential conflicts of interest.*** All authors: No reported conflicts. All authors have submitted the ICMJE Form for Disclosure of Potential Conflicts of Interest. Conflicts that the editors consider relevant to the content of the manuscript have been disclosed.
