# Supplementary material for: Design and Analysis of Elimination Surveys for Neglected Tropical Diseases
Source: J Infect Dis. 2020 Jan 13;221(Suppl 5):S554–60. doi: 10.1093/infdis/jiz554 (PMC7289555; doi:10.1093/infdis/jiz554)
Supplement: jiz554_suppl_Supplemental_Material [file jiz554_suppl_supplemental_material.pdf]

Supplementary material
Click here to access/download;Manuscript;supplementary_material.tex

Supplementary Material

1 The geostatistical model for prevalence mapping

A formal definition of the geostatistical model used in this paper, in which we make explicit the role of the model parameters, is the following.

1. $S(x) : x \in A$ is a stationary Gaussian process with mean zero, variance σ^2 and correlation function $\rho(u/\phi)$, where u denotes distance.
2. $Z(x)$ is uncorrelated Gaussian white noise with mean zero and variance ν^2.
3. Conditional on $S(\cdot)$ and $Z(\cdot)$, the numbers, Y_i, of positive test results are independent, binomially distributed with binomial denominators m_i and binomial probabilities $P(x_i)$ defined by the following equation

$$\log[P(x)/\{1 - P(x)\}] = \alpha + d(x)'\beta + S(x) + Z(x). \quad (1)$$

In the above definition, the parameter ϕ determines the *range* of the spatial correlation in the log-odds of prevalence whilst the two variance components σ^2 and ν^2 determine its strength. The correlation between log-odds of prevalence at two locations a distance u apart is

$$r(u) = \sigma^2\rho(u/\phi)/(\sigma^2 + \nu^2). \quad (2)$$

The conditional binomial distribution for the Y_i follows from elementary probability theory.

1.1 Parameter estimation

For the geostatistical model defined above, we estimate the model parameters $\theta = (\alpha, \beta, \sigma, \phi, \nu)$ by Monte Carlo maximum likelihood (Geyer and Thompson, 1992; Christensen, 2004). Maximum likelihood is a generally efficient method of estimation, whilst its Monte Carlo version extends its applicability to models whose likelihood functions are intractable. A general property of maximum likelihood estimators, $\hat{\theta}$, is that in large samples they are approximately unbiased and multivariate Normally distributed with a variance matrix, Σ, that can also be estimated as a by-product of the maximisation algorithm for finding $\hat{\theta}$. In the current context we apply the Monte Carlo maximum likelihood method to a transformed set of parameters to improve the multivariate Normal approximation (Diggle and Giorgi, 2019).

As with any numerical optimisation method, Monte Carlo maximum likelihood needs to be handled with care. In particular, it relies on the user providing reasonable starting values for

the covariance parameter estimates σ^2, ϕ and ν^2. A useful tool for this is the *variogram*. The variogram, $V(u)$, of a stationary spatial stochastic process is defined to be one half of the variance of the difference between values of the process at locations a distance u apart. For our model, the variogram of the log-odds of prevalence is

$$V(u) = \nu^2 + \sigma^2\{1 - \rho(u/\phi)\}. \tag{3}$$

The left-hand panel of Figure S1 gives a schematic picture of a generic variogram whilst the right-hand panel shows an estimated variogram for data on LF prevalence in Ghana, which we will analyse in our case-study. The shaded area on the estimated variogram covers the range of estimated variograms under repeated random permutations of the empirical logit-transformed prevalence values amongst the sampled locations. This represents the sampling distribution of the estimated variogram in the absence of spatial correlation, and confirms the presence of spatial correlation in the data with a range of very roughly 100km. We choose the shape of the correlation function and initial values for the model parameters by matching the generic form to the estimate; a "by eye" matching is usually good enough and is facilitated by the `eyefit()` function in the `PrevMap` package.

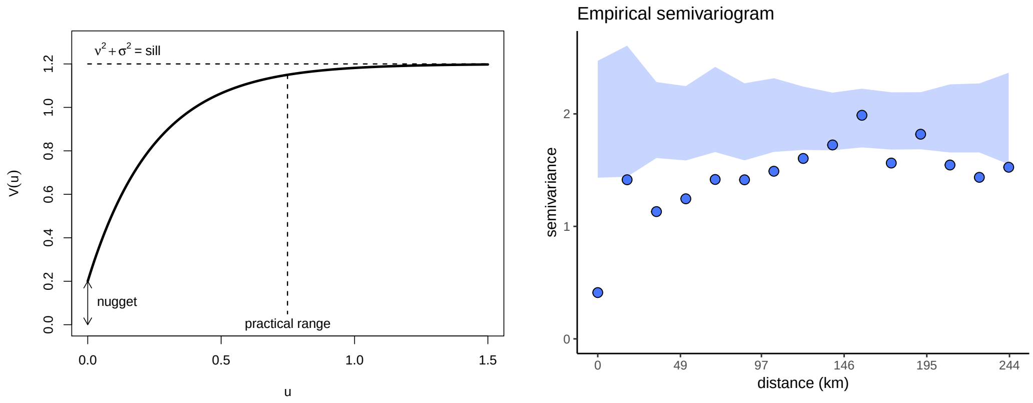

Figure S1: Left-hand panel: the generic form of the variogram, $V(u)$. The *nugget* is another name for the sampling variance of the response variable (in the current context, the empirical-logit-tranformed prevalence). The *sill* is another name for total variance of the response variable; the difference between the sill and the nugget is the variance of the spatially correlated conmponent of variance. The *practical range* is the distance u at which the spatial correlation function $\rho(u)$ decays to 0.05. Right-hand panel: an estimated variogram calculated from the data on lymphatic filariasis prevalence in Ghana. The shaded area covers the range of estimated variograms under repeated random permutations of the empirical logit-transformed prevalence values among the sample locations.

1.2 Spatial prediction

For prevalence mapping, interest typically lies not in the parameters themselves but in what they tell us about the prevalence surface $\mathcal{P} = \{P(x_j^*) : j = 1, ..., N\}$. As noted above, the

predictive distribution of $\mathcal{P}$ is its conditional distribution given the data. For plug-in prediction, we draw samples from this conditional distribution with the elements of θ held fixed at their Monte Carlo maximum likelihood estimates. This ignores parameter uncertainty. In many cases the effects of parameter uncertainty are negligible relative to the predictive uncertainty in $P(x)$, because all of the data contribute information about θ whereas only data from locations relatively close to x contribute information about $P(x)$. Nevertheless, to account for parameter uncertainty we can draw samples from the conditional distribution of $\mathcal{P}$ given θ, in which the value of θ for each sample is itself drawn from a multivariate Normal distribution with mean $\hat{\theta}$ and variance matrix $\hat{\Sigma}$.

The sampled prevalence surfaces, $\{\mathcal{P}_k : k = 1, ..., s\}$ say, from the predictive distribution of $\mathcal{P}$ can then be used to make probability statements about the underlying true prevalence surface, or any of its properties. Formally, if $T = \mathcal{T}(\mathcal{P})$ is any predictive target, then $\{T_k = \mathcal{T}(\mathcal{P}_k) : k = 1, ..., s\}$ is a sample from the predictive distribution of T.

Reasonable choices for a "best guess" map of prevalence over the area of interest would be the sample mean or median of the sampled values of prevalence at each location. If, as is typically the case in elimination surveys, the primary objective is to assess whether a pre-specified prevalence theshold has been met, we advocate mapping the *predictive exceedance probability*, i.e. the probabilty for each location or area of interest that prevalence exceeds the pre-specified threshold. In the absence of a pre-declared threshold, we recommend constructing a sequence of such maps over a range of prevalence thresholds.

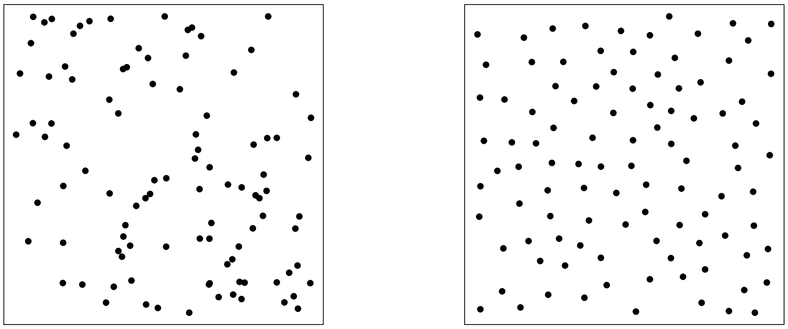

Figure S2: A set of 100 random data-locations (left-hand panel) and a more efficient, spatially regulated configuration of 100 data-locations (right-hand panel) placed randomly subject to the constraint that no two of them may be separated by a distance less than $\delta = 0.07$

2 Model fitted to baseline data

We fitted the geostatistical model described in the methods section to baseline LF prevalence data shown in Figure S3, using Monte Carlo maximum likelihood. The resulting parameter

estimates with their respective 95% confidence intervals are reported in Table 1. The scale parameter ϕ can be more easily interpreted in terms of the corresponding *practical range*, defined as the distance at which the spatial correlation is approximately 0.05. The estimated *practical range* is 95.965 $(62.913, 146.383)$ km. This model includes no covariates.

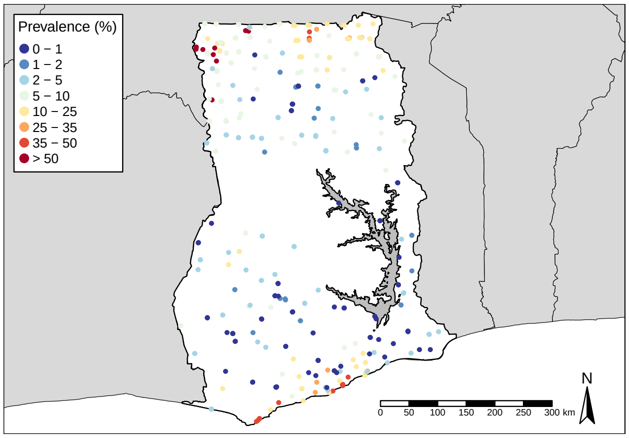

Figure S3: Locations and empirical prevalences from 403 LF prevalence surveys in Ghana (data extracted from WHO ESPEN portal `http://espen.afro.who.int/`).

Table 1: Monte Carlo maximum likelihood estimates and corresponding 95% confidence intervals for the model fitted to LF pre-intervention data.

Parameter	Estimate	95% CI
$\hat{\alpha}$	-3.390	(-3.897, -2.882)
$\hat{\sigma}^2$	2.695	(1.897, 3.828)
$\hat{\phi}$	32.034	(21.001, 48.864)
$\hat{\nu}^2$	0.168	(0.072, 0.393)

Figure S4 summarises the predicted prevalence surface at baseline.

3 Emulating the TAS sampling design

3.1 Determining the eligibility of an EU

A pre-TAS survey is conducted in each EU after completion of five rounds of MDA with populaton coverage greater than 65% (WHO, 2011). If EU prevalence is less than 2% in all sentinel and spot-check sites then the EU is eligible for the TAS. For our analysis, we sampled three sentinel sites at random in each EU and classified an EU as eligible or not for the TAS accordingly. This resulted in 164 EUs being declared eligible for TAS (see Figure S5) and therefore included in our simulations.

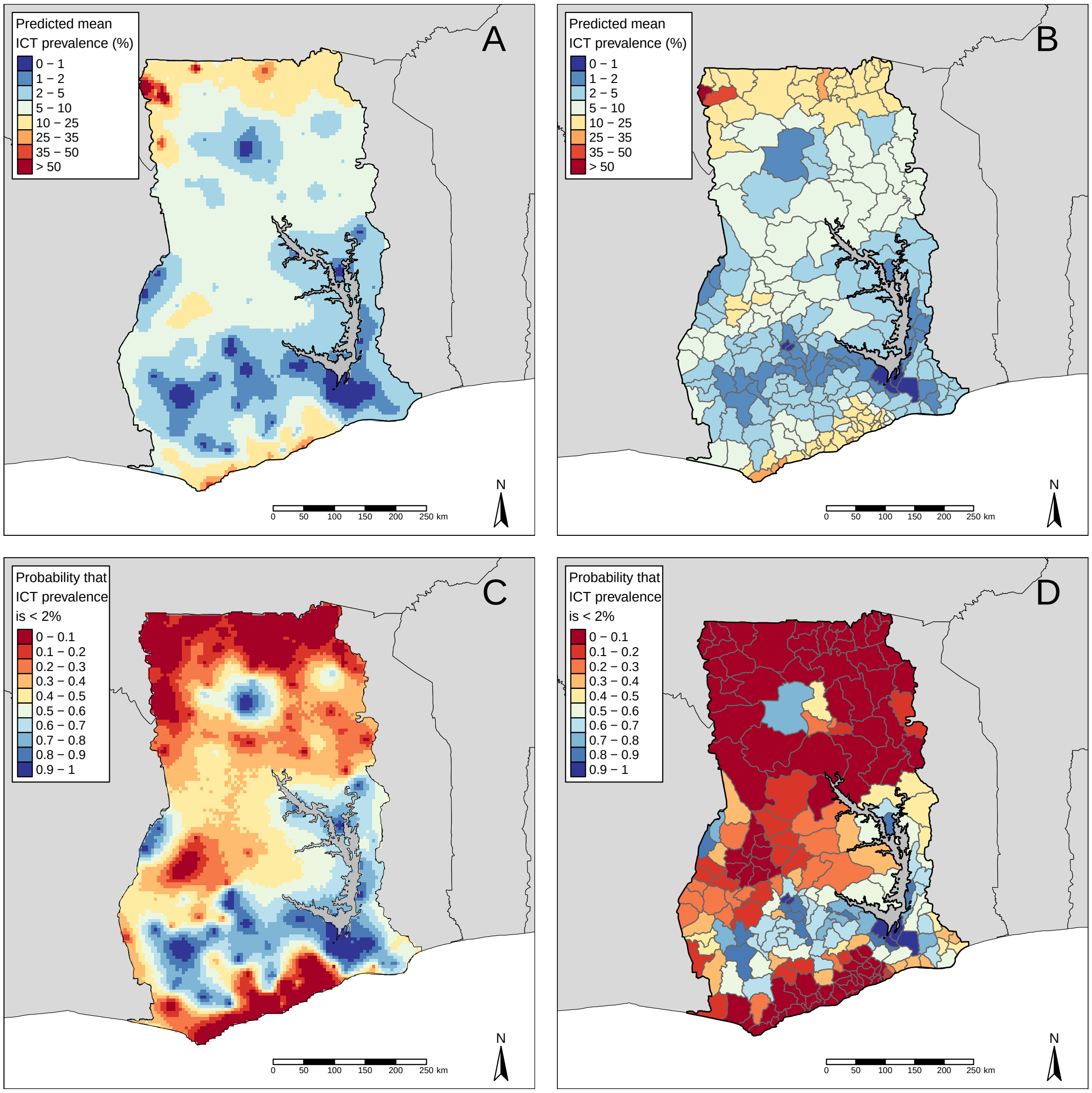

Figure S4: Baseline predicted ICT mean prevalence at 5km pixel level (A); baseline predicted mean population weighted ICT prevalence at EU level (B); probability map for non-exceedance of 2% prevalence at pixel level (C); probability map for non-exceedance of 2% population weighted ICT prevalence at EU level (D).

3.2 Estimating the numbers of six to seven year old children

For the analyses reported in the paper, we treat each district as an EU. We downloaded population estimates for Ghana in five-year age bands at a resolution of 100m from WorldPop (`www.worldpop.org`). We multiplied the six-to-ten year old population raster by 2/5 and summed all pixels inside each EU to obtain an estimate of the total number of six-to-seven year old children (Figure S6).

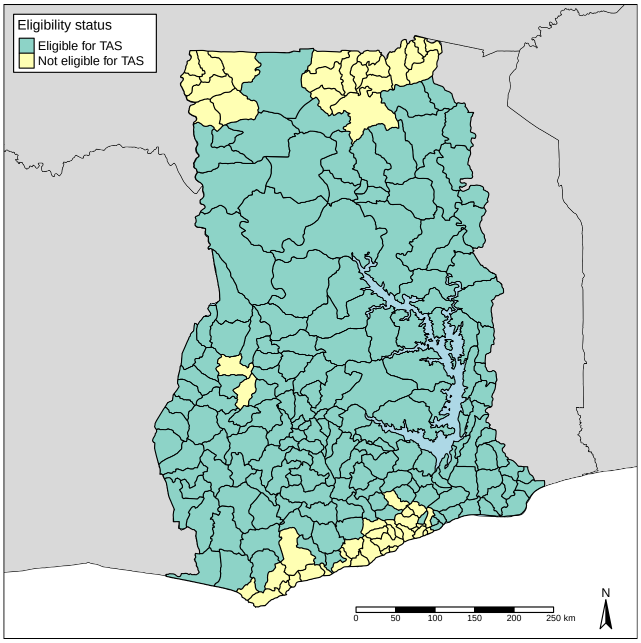

Figure S5: Eligibility of Ghana EUs for the TAS.

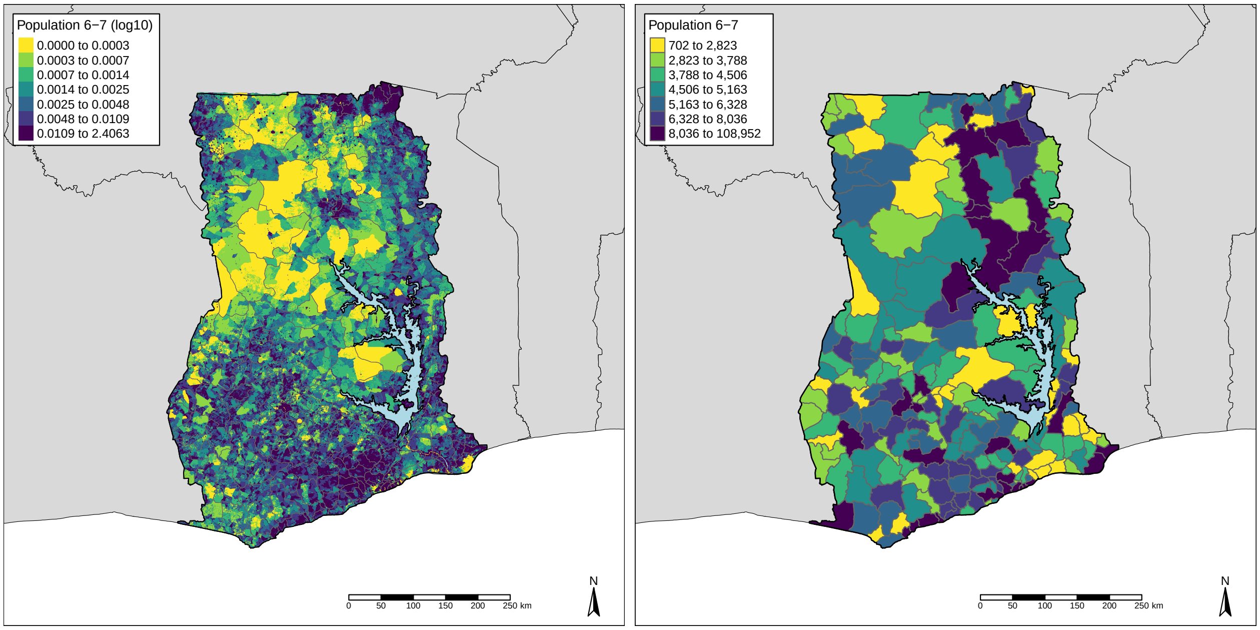

Figure S6: Population estimates (on the log10 scale) of 6-7 years old at pixel level (left), population estimates of 6-7 years old children at EU level (right).

3.3 Villages to be sampled

Figure S7 shows the locations of villages eligible to be sampled. Their locations were retrieved by combining data from OpenStreetMap (`https://www.openstreetmap.org/`) and Geonames (`http://www.geonames.org`).

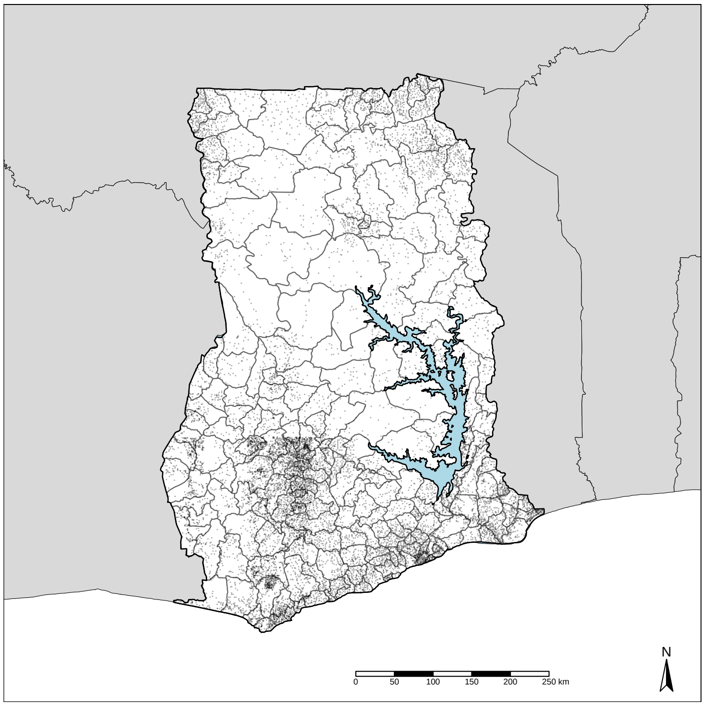

Figure S7: Spatial distribution of villages eligible for the TAS.

3.4 Assessing the TAS survey design parameters

Using the information provided by Table A.5.2 in WHO (2011) and the estimated populations of six-to-seven year old children in each EU, we selected the design parameters to emulate the TAS. These are, for each EU: the total number of children to be tested; the number of clusters (villages or schools) to be sampled; the critical cut-off (maximum number of children testing positive to declare elimination).

References

Christensen, O. F. (2004). Monte carlo maximum likelihood in Model-Based geostatistics. *J. Comput. Graph. Stat.*, 13(3):702–718.

Diggle, P. J. and Giorgi, E. (2019). *Model-based Geostatistics for Global Public Health: Methods and Applications.* CRC Press.

Geyer, C. J. and Thompson, E. A. (1992). Constrained monte carlo maximum likelihood for dependent data. *J. R. Stat. Soc. Series B Stat. Methodol.*, 54(3):657–683.

WHO (2011). *Monitoring and epidemiological assessment of mass drug administration in global programme to eliminate lymphatic filariasis: a manual for national elimination programmes.* World Health Organization.
